# Differential effects of type 2 diabetes and gestational diabetes on maternal and cord blood adipokines and newborn weight

**DOI:** 10.1186/s12884-025-07169-z

**Published:** 2025-03-05

**Authors:** Brittany L. Gruber, Yash Rawal, Priscilla Irabor, Elizabeth A. C. Sellers, Christy Pylypjuk, Vernon W. Dolinsky, Brandy A. Wicklow

**Affiliations:** 1 Department of Pharmacology & Therapeutics, University of Manitoba, 601 John Buhler Research Centre 715 McDermot Ave, Winnipeg, MB R3E 3P4 Canada; 2 Department of Pediatrics & Child Health, University of Manitoba, Manitoba Clinic, Level 7- 820 Sherbrooke St., Winnipeg, MB R3A 1R9 Canada; 3 Department of Obstetrics & Gynecology, University of Manitoba, 513 John Buhler Research Centre 715 McDermot Ave, Winnipeg, MB R3E 3P4 Canada; 4 Diabetes Research Envisioned and Accomplished in Manitoba (DREAM) Research Theme of the Children’s Hospital Research Institute of Manitoba, Winnipeg, MB Canada

**Keywords:** Pregnancy, Gestational diabetes, Type 2 diabetes, Fetal growth, Adiponectin, Leptin

## Abstract

**Background:**

Dysregulated adipokine levels are associated with type 2 diabetes and gestational diabetes. Adiponectin and leptin are involved in nutrient transport, thereby affecting fetal growth and metabolism. We aimed to determine whether type 2 diabetes and gestational diabetes were associated different levels of serum and cord blood adiponectin, leptin, insulin and offspring birthweight.

**Methods:**

Serum, cord blood, gestational age and birthweight were collected for First Nations mothers and infants who were enrolled in the Next Generation Cohort Study. A total of 173 maternal and 188 neonatal samples were available for analysis. Of those, 136 were matched maternal infant dyads that we used for paired mother-infant analyses. Pairs were sorted into groups based on maternal diagnoses of pre-existing type 2 diabetes, gestational diabetes or no diabetes (control). Adiponectin and leptin were measured by enzyme linked immunosorbent assay.

**Results:**

Mothers with gestational diabetes had lower serum adiponectin (6.48 ± 3.64 µg/mL) in the third trimester relative to mothers with type 2 diabetes (8.55 ± 5.24 µg/mL, *p* < 0.05) or no diabetes (7.73 ± 3.47 µg/mL). However, cord blood adiponectin was lower only in normal weight pregnancies complicated by type 2 diabetes. Cord blood glucose, insulin and leptin were increased in infants of type 2 diabetes mothers and increased leptin was positively correlated with maternal leptin and birth weight. Female infants exposed to pregestational type 2 diabetes had a significantly higher birthweight z-score than female control infants.

**Conclusions:**

In this study, exposure to type 2 diabetes, but not gestational diabetes, impacted cord blood levels of glucose, insulin and leptin and birthweight. Collectively, these factors may contribute to the greater impact of pregestational type 2 diabetes exposure on offspring health relative to gestational diabetes.

**Supplementary Information:**

The online version contains supplementary material available at 10.1186/s12884-025-07169-z.

## Background

Diabetes during pregnancy is increasingly common and strongly associated with maternal obesity [1]. Increasing numbers of women are being diagnosed prior to pregnancy with type 2 diabetes (T2D) and during pregnancy with gestational diabetes mellitus (GDM) [2]. Worldwide the prevalence of GDM is approximately 14%, and pre-existing T2D is approximately 0.2% [3, 4]. Offspring born to mothers with diabetes during pregnancy are more likely to be large for gestational age and are at increased risk complications during labour and delivery [5]. Offspring exposed to diabetes during pregnancy are also more likely to develop obesity and metabolic syndrome later in life [6, 7].

While T2D and GDM share some similar risk factors, there is some evidence that the health outcomes for women and their offspring are different. Women with T2D have a higher risk of preeclampsia, C-section and operative vaginal delivery, and a higher incidence renal disease relative to women with GDM or no diabetes in pregnancy [8, 9]. Exposure to T2D in utero has a more profound effect on the earlier diagnosis of T2D in the offspring compared to GDM [10, 11]. This suggests developmental programming effects that alter growth, metabolism, and increased obesity risk in the offspring.

Adipokines are adipose tissue derived hormones which regulate insulin sensitivity and metabolism. Adiponectin is abundant in serum and increases the insulin sensitivity of metabolic tissues. In a healthy pregnancy, adiponectin decreases modestly towards the end of gestation in parallel with increasing gestational weight gain and the physiological insulin resistance of pregnancy that spares glucose for the fetus [12]. GDM is associated with a further reduction of circulating adiponectin [12]. In contrast, leptin increases throughout gestation. Obese women have higher serum levels of leptin compared to women with a lower body mass index (BMI) in pregnancy [13]. Independent of BMI, women with GDM have increased levels of serum leptin and there is a positive association between serum leptin, serum insulin and insulin resistance, suggesting these factors are linked in GDM [14]. However, similar data does not presently exist for pregestational T2D.

Levels of adipokines in the maternal and fetal system are also implicated in fetal growth and metabolism. Maternal adiponectin levels generally show an inverse relationship with birthweight [15]. In contrast, a positive association of fetal growth and adiposity with fetal adiponectin has been reported [16]. Fetal leptin, produced by fetal adipose tissue, increases throughout gestation [17] and is positively associated with cord-blood leptin and birthweight [18], indicating that leptin also has a role in growth and metabolism.

Previous work by our group [10] demonstrated that infants exposed to pregestational T2D had higher rates of both large- and small-for-gestation age compared to infants exposed to GDM [10]. Since dysregulation of maternal and fetal adipokines are linked to fetal growth and adiposity, the objective of this study was to determine whether the timing of exposure to diabetes during pregnancy (GDM versus pre-gestational T2D) impacts adiponectin, leptin and insulin levels in maternal serum and/or cord blood and whether these levels were associated with birthweight.

## Methods

### Subjects

We obtained maternal and cord blood serum from 136 First Nations mother and infant dyads enrolled in the Next Generation cohort in Manitoba, Canada. A detailed description of this cohort has previously been published [19]. Women without diabetes in pregnancy (control), with GDM or T2D, of self-declared First Nations heritage who were delivering and residing in Manitoba were included.

### Population characteristics and anthropometric measurements

Population characteristics and anthropometric values for mothers and babies were obtained from a combination of self-reported data (ethnicity, smoking status during pregnancy and medical history) and hospital records. Ponderal index was calculated from neonatal birthweight and length. Birthweight and length z-scores and percentiles were calculated against the International Fetal and Newborn Growth Consortium for the 21st Century (INTERGROWTH 21st) calculator and database, accounting for sex and gestational age.

### Serum collection

Maternal serum and cord blood were collected at the time of delivery for analysis of adiponectin and leptin levels. Samples were collected in 4mL EDTA and 5 mL SST tubes, respectively. Serum was separated from red blood cells by centrifugation (2600 rpm for 15 min at 4 °C), aliquoted and then stored at -80 °C.

### Serum measurements

Adiponectin and leptin concentrations in maternal and cord blood serum were determined using human adiponectin and leptin ELISAs according to manufacturer’s instructions (Human Adiponectin ELISA and Human Leptin ELISA, ALPCO, Salem, NH, USA). Hemoglobin A1c (HbA1c) concentrations and cord blood insulin were analysed on a Roche Cobas (Roche Diagnostics (Schweiz), Rotkreuz, ZUG, Switzerland).

### Statistical analysis

Data was analyzed for normal distribution using Shaprio-Wilk test. Data that met assumptions of normal distribution were analyzed with one- or two-way analysis of variance (ANOVA) to compare group means, and post-hoc tests for multiple comparisons. Data that were not normally distributed were analyzed with Kruskal-Wallis non-parametric test and, where appropriate, Dunn post-hoc test for multiple comparisons. When comparing two groups, normally distributed data were analyzed with a t-test, and non-normally distributed data were analyzed with Mann-Whitney U non-parametric test. Correlations are represented by the Spearman correlation coefficient for non-normally distributed data and represented by r value and significance denoted by p-value. P-values calculated from Spearman correlation are approximate when comparing 18 or more pairs, and exact when comparing 17 or fewer. Statistical significance is defined as *p* < 0.05. Analysis was performed using GraphPad Prism Software (La Jolla, CA, USA).

## Results

### Maternal characteristics

Serum from 136 mother-infant pairs was analyzed. Of these pairs, 57 mothers had pregestational T2D, 49 had GDM and the control group comprised of 30 infants and normoglycemic mothers. HbA1c was highest in mothers with T2D, (mean 7.54%) indicating suboptimal glucose control in the third trimester (Supplemental Table 1). A diabetes diagnosis in pregnancy was associated with stepwise increases in HbA1c; mothers with T2D had significantly higher HbA1c (*p* < 0.0001 vs. GDM, *p* < 0.0001 vs. control) than mothers with either GDM or no diabetes, and mothers with GDM had significantly higher HbA1c than mothers with no diabetes (*p* = 0.003) (Supplemental Table 1). In the T2D group, maternal HbA1c was negatively correlated with gestational age (Table 1). In addition, HbA1c levels in the maternal T2D group were correlated with birthweight z-scores (Table 1). Maternal age at delivery did not differ between the groups (Supplementary Table 1). At enrollment, the BMI of GDM and T2D mothers were similar to control mothers. However gestational weight gain was the least in mothers with GDM relative to control (*p* = 0.024) and T2D mothers (*p* = 0.014) (Supplementary Table 1). Mothers with GDM or T2D also had higher parity (*p* = 0.001 GDM vs. control, *p* = 0.041 T2D vs. control) and gravidity (*p* = 0.005 GDM vs. control, *p* = 0.024 T2D vs. control) compared to control mothers. There were no significant differences in these parameters between T2D and the control and GDM groups.

**Table 1:** Correlations between maternal adipokines and neonatal outcomes in paired mother-infant dyads from control, GDM and T2D pregnancies. Values represent Spearman’s correlation coefficient (r value) from regressions performed on maternal and cord blood adipokine and serum measures against maternal and fetal anthropometric values. Bold font indicates statistical significance (*p* < 0.05) determined from Spearman correlation analysis. Italic indicates approximate p-value, computed for non-parametric correlation with > 18 pairs. BW, birthweight; GDM, gestational diabetes mellitus; T2D, type 2 diabetes mellitus; APN, adiponectin; LEP, leptin; GA, gestational age; BMI, body mass index; HbA1c, hemoglobin A1c

	Control				GDM				T2D			
	Maternal APN	Maternal LEP	GA (weeks)	BW z-score	Maternal APN	Maternal LEP	GA (weeks)	BW z-score	Maternal APN	Maternal LEP	GA (weeks)	BW z-score
Maternal APN		-0.141	**-0.509** (*p* = 0.009)	-0.371		0.082	0.269	**-0.370** (*p* = 0.012)		**-0.321** (*p* = 0.019)	0.025	**-0.286** (*p* = 0.047)
Maternal Leptin			0.261	0.217			0.127	0.076			-0.159	-0.148
Maternal Blood Glucose	-0.073	0.538	-0.164	0.328	-0.044	0.458 (*p* = 0.042)	-0.224	0.025	-0.032	0.117	-0.025	-0.033
Maternal HbA1c	-0.229	-0.021	0.363	0.247	0.070	0.222	-0.151	-0.048	0.058	0.147	-**0.458**(*p* = 0.001)	**0.386** (*p* = 0.008)
BMI at Delivery	**-0.545** (*p* = 0.007)	0.409	**0.414** (*p* = 0.044)	**0.497** (*p* = 0.016)	-0.253	**0.358**(*p* = 0.018)	-0.161	0.150	-0.037	0.207	-0.113	0.091

### Maternal serum adipokines

**Fig. 1:**
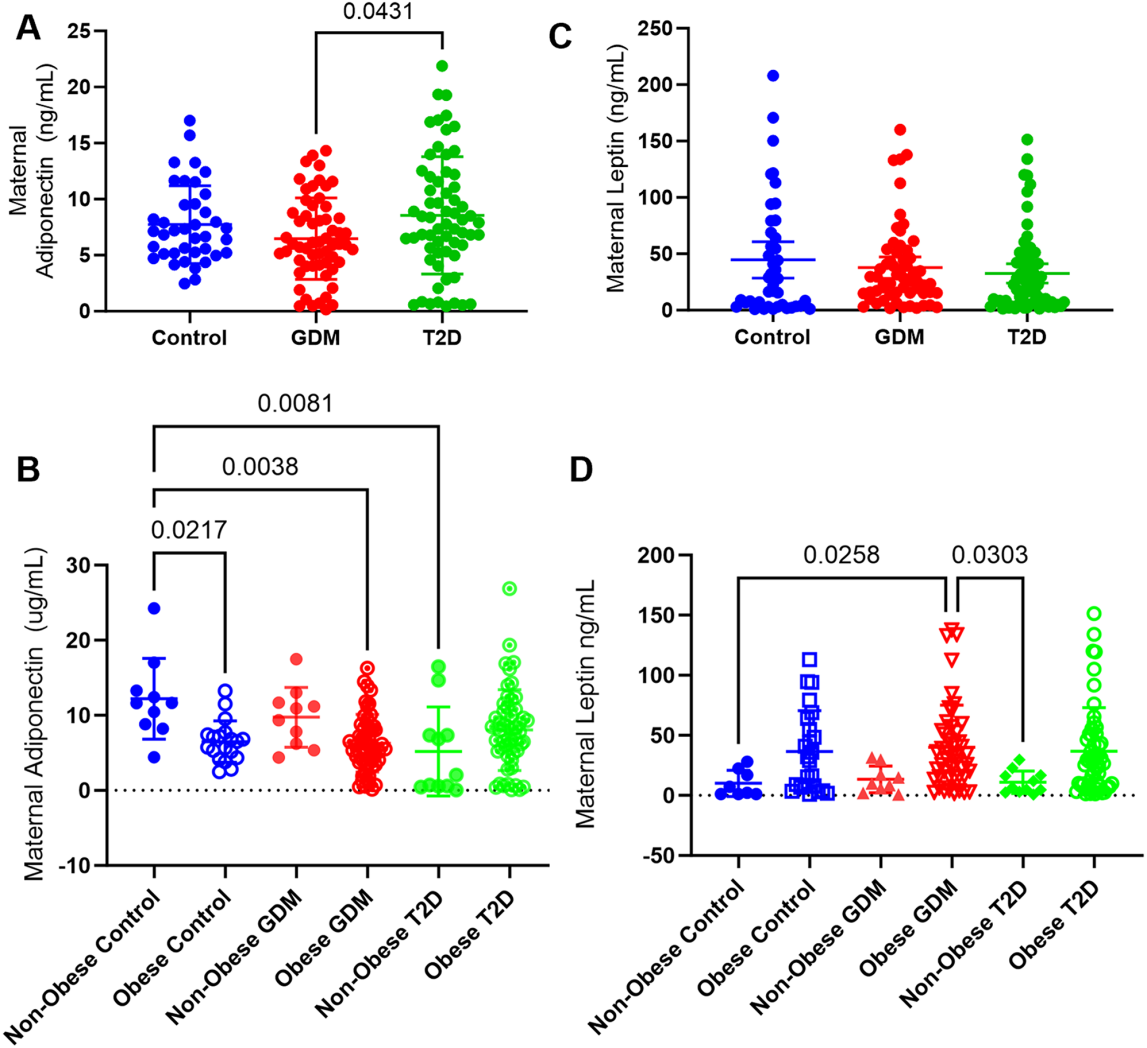
Maternal serum adipokines. Maternal serum adiponectin (**A**) from all control mothers (*n* = 39), mothers with GDM (*n* = 59) and T2D (*n* = 63) Maternal adiponectin (**B**) from all mothers separated by coexistence of maternal obesity (BMI > 30 kg/m2), non-obese control (*n* = 10), obese control (*n* = 19), non-obese GDM (*n* = 10), obese GDM (*n* = 50), non-obese T2D (*n* = 11), and obese T2D (*n* = 52). Maternal serum leptin (**C**) from all control mothers (*n* = 42), mothers with GDM (*n* = 59), or T2D (*n* = 69). Maternal serum leptin from all mothers separated by coexisting obesity (**D**), non-obese control (*n* = 8), obese control (*n* = 24), non-obese GDM (*n* = 9), obese GDM (*n* = 47), non-obese T2D (*n* = 11), and obese T2D (*n* = 52)

Overall, mothers with GDM had significantly (*p* = 0.0431) lower serum adiponectin compared to mothers with T2D (Fig. 1A). Between all mothers with obesity, there were no significant differences in maternal serum adiponectin. Compared to non-obese mothers without diabetes, obese mothers without diabetes (*p* = 0.022), obese mothers with GDM (*p* = 0.0038) and non-obese mothers with T2D (*p* = 0.008) also had lower serum adiponectin (Fig. 1B). Maternal serum leptin levels were similar across all control, GDM and T2D pregnancies (Fig. 1C). When stratified by BMI, maternal leptin was higher in obese mothers with GDM compared to non-obese mothers without diabetes (*p* = 0.026), and non-obese mothers with T2D (0 = 0.030) (Fig. 1D). Maternal serum adiponectin did not show a correlation with maternal serum leptin in control or GDM mothers but was negatively correlated (*p* = 0.025) in mothers with T2D (Table 1).

Maternal adiponectin showed a strong negative correlation (*r*=-0.545, *p* = 0.007) with maternal BMI at delivery in control pregnancies, but not GDM or T2D pregnancies (Table 1). A strong negative correlation between maternal adiponectin and gestational age (*r*=-0.509, *p* = 0.009) was observed in control, but not in the GDM and T2D pregnancies (Table 1). In addition, a weaker negative correlation between maternal adiponectin and birthweight z-score was observed in GDM pregnancies (*r*=-0.370, *p* = 0.012), and T2D pregnancies (*r*=-0.286, *p* = 0.047) but not control pregnancies (Table 1).

Maternal serum adiponectin had a negative correlation with maternal CRP levels (*p* = 0.032) in the T2D mothers (Supplemental Table 3), but not controls or GDM mothers. Maternal serum leptin was positively correlated with maternal CRP (*r* = 0.517, *p* = 0.006) in the control group (Supplemental Table 3), but not the GDM or T2D pregnancies.

Maternal serum leptin showed a weakly positive correlation with maternal BMI at delivery in GDM pregnancies (*r* = 0.319, *p* = 0.018), but not control or T2D pregnancies (Table 1). Maternal serum leptin did not associate with gestational age or birthweight z-score in any pregnancies (Table 1). A negative association was observed between maternal leptin and parity only among the control mothers (*r*= -0.477. *p* = 0.021) (Supplemental Table 3).

### Neonatal characteristics

Birthweight was expressed as z-score using the International Fetal and Newborn Growth Consortium for the 21st Century (INTERGROWTH-21st ) database, accounting for sex and gestational age. In control mothers, birthweight z-score was moderately positively correlated with BMI at delivery (*r* = 0.497. *p* = 0.016) (Table 1). Female infants exposed to pregestational T2D were significantly larger than female control infants (*p* = 0.0001), and female infants from GDM mothers (*p* = 0.031) (Supplemental Table 2). Females from T2D mothers had a higher birthweight z-score than male infants from T2D mothers (*p* = 0.002) (Supplemental Table 2). There were no significant differences in length z-score of female or male infants between groups. Ponderal index considers the length and birthweight and is a measure of overall growth [20] and female infants exposed to pregestational T2D had significantly higher ponderal index relative to infants from control mothers (*p* = 0.002) and were also larger than male infants exposed to pregestational diabetes (*p* = 0.003) (Supplemental Table 2). Infants born to mothers with GDM or T2D were born at a significantly younger gestational age relative to control infants (Supplemental Table 2). When adjusted for neonatal sex, gestational age of both male and female infants was lower in the GDM (male *p* = 0.004, female *p* = 0.004) and T2D groups (male *p* = 0.0002, female *p* < 0.0001) compared to controls (Supplemental Table 2).

### Infant cord blood adipokines

**Fig. 2:**
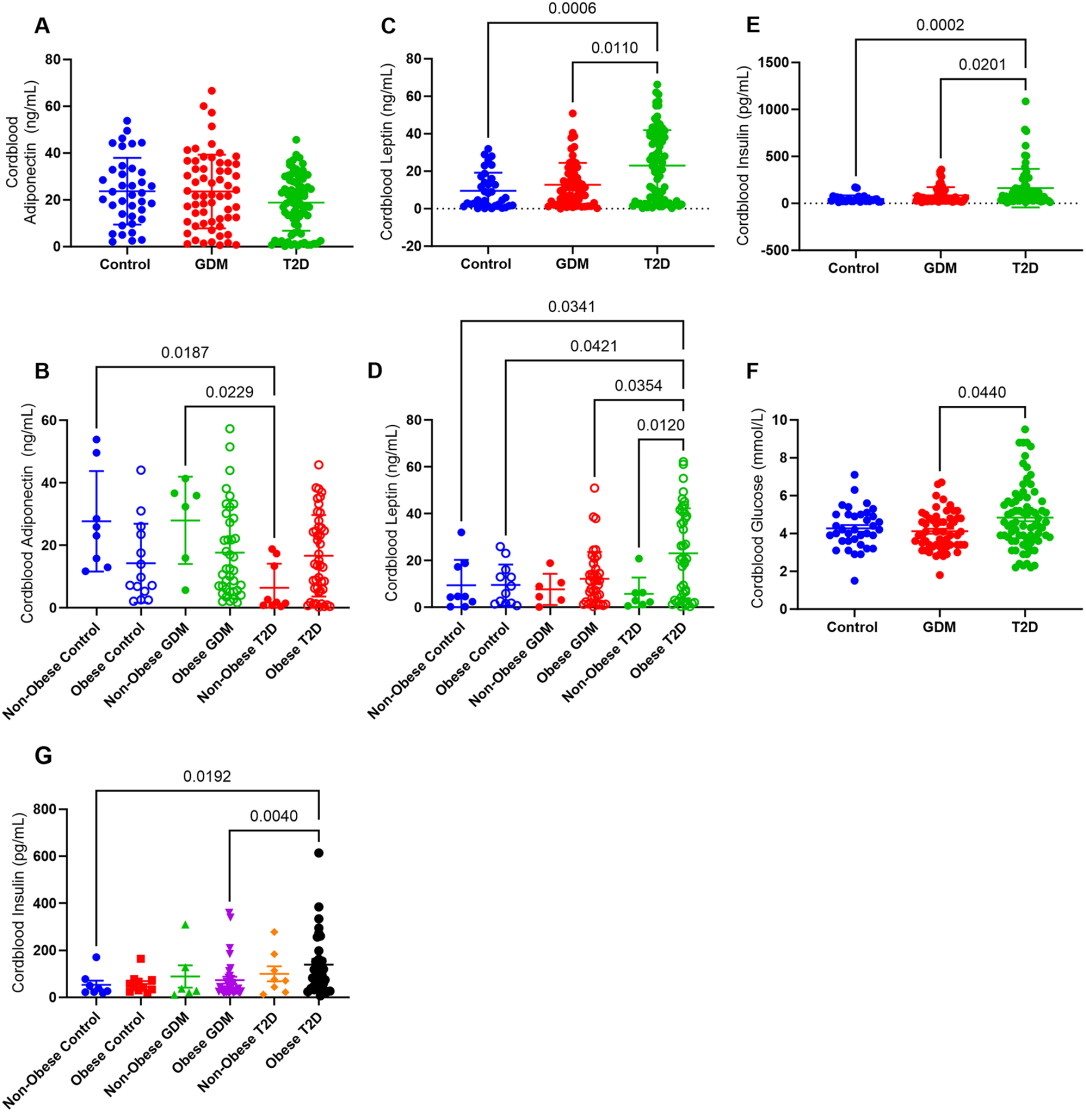
Cord-blood adipokines, insulin and glucose at delivery. Cord-blood adiponectin (**A**) from neonates born to all control mothers (*n* = 38), GDM (*n* = 61) or pre-gestational T2D (*n* = 83). Cord-blood adiponectin from maternal-infant dyads separated by BMI (**B**), non-obese control (*n* = 9), obese control (*n* = 14), non-obese GDM (*n* = 6), obese GDM (*n* = 39), non-obese T2D (*n* = 9) and obese T2D (*n* = 44). Cord-blood leptin (**C**) from neonates born to all control mothers (*n* = 35), GDM (*n* = 60), and T2D (*n* = 83). Cord-blood leptin from maternal-infant dyads separated by maternal BMI (**D**), non-obese control (*n* = 9), obese control (*n* = 12), non-obese GDM (*n* = 6), obese GDM (*n* = 39), non-obese T2D (*n* = 7), and obese T2D (*n* = 42). Cord-blood insulin (**E**) from all neonates born to control mothers (*n* = 23), GDM (*n* = 43), and T2D (*n* = 59). Cord-blood glucose levels in neonates born to control mothers (*n* = 36), GDM (*n* = 57) and T2D (*n* = 76). G) Cord-blood insulin from paired maternal-infant dyads, striated by maternal obesity Non-obese control *n* = 8, obese control *n* = 11, non-obese GDM *n* = 6, obese GDM *n* = 33, non-obese T2D *n* = 8, obese T2D *n* = 32)

Cord blood adiponectin was similar between infants of control, GDM and T2D pregnancies (Fig. 2A). In offspring born to mothers with BMI < 30 kg/m^2^ (non-obese) cord blood adiponectin was lower in those exposed to T2D relative to control mothers (*p* = 0.019) and mothers with GDM (*p* = 0.023) (Fig. 2B). Cord blood leptin from all infants of T2D pregnancies was significantly higher than those from control (*p* = 0.0006) and GDM (*p* = 0.0110) pregnancies (Fig. 2C). Infants born to mothers with both T2D and obesity had significantly higher cord blood leptin than those exposed to only T2D in pregnancy (*p* = 0.012), and higher cord blood leptin than infants exposed to both GDM and obesity in pregnancy (*p* = 0.035) (Fig. 2D). The cord blood insulin of infants of T2D mothers was markedly increased relative to GDM pregnancies (*p* = 0.020) and control pregnancies (*p* = 0.0002) (Fig. 2E). Cord blood insulin was higher in obese T2D pregnancies than obese GDM pregnancies (*p* = 0.004), and non-obese control pregnancies (*p* = 0.0192), but there was no difference in the absence of obesity (Fig. 2G). Infants born to mothers with T2D showed a sex specific difference in cord blood insulin levels, with females having significantly higher cord blood insulin compared to males (*p* = 0.034) (Supplemental Fig. 1). Cord blood glucose did not vary based on neonatal sex, but cord blood glucose levels were significantly higher in neonates born to mothers with T2D relative to mothers with GDM (*p* = 0.044) (Fig. 2F), and this appears to be limited to pregnancies affected by obesity (*p* = 0.012) (Supplemental Fig. 1).

Cord blood adiponectin showed a positive correlation with maternal adiponectin in GDM (*p* = 0.008) and T2D (*p* < 0.0001) pregnancies (Table 2), but not control pregnancies. Cord blood adiponectin was strongly positively associated with cord blood leptin in infants of control pregnancies (*r* = 600, *p* = 0.001) and in infants of T2D (*p* = 0.012) pregnancies, but not infants from GDM pregnancies (Table 2). Cord blood adiponectin was also positively correlated with maternal serum leptin (*p* = 0.012) in control and GDM (*p* = 0.016) pregnancies but showed no correlation in T2D pregnancies (Table 2).

**Table 2:** Correlations between cord blood adipokines and maternal characteristics and neonatal outcomes in paired mother-infant dyads from control, GDM and T2D pregnancies. Values represent Spearman’s correlation coefficient (r value) from regressions performed on maternal and cord blood adipokine and serum measures against fetal anthropometric values. Bold font indicates statistical significance (*p* < 0.05) determined from Spearman correlation analysis. Italic indicates approximate p-value, computed for non-parametric correlation with > 18 pairs. CB, cord-blood; BW, birthweight; GDM, gestational diabetes mellitus; T2D, type 2 diabetes mellitus; APN, adiponectin; LEP, leptin; GA, gestational age; BMI, body mass index; HbA1c, hemoglobin A1c

	Control				GDM				T2D			
	CB APN	CB Leptin	BW z-score	CB Insulin	CB APN	CB Leptin	BW z-score	CB Insulin	CB APN	CB Leptin	BW z-score	CB Insulin
CB APN			0.187				-0.005				-0.135	
CB Leptin	**0.600**(*p* = 0.001)		**0.402** (*p* = 0.044)		0.278		**0.319**(*p* = 0.031)		**0.342**(*p* = 0.012)		0.176	
CB Insulin	0.331	**0.443** (*p* = 0.044)	**0.696**(*p* < 0.0001)		-0.267	0.173	**0.334**(*p* = 0.038)		0.096	0.246	**0.743** (*p* < 0.0001)	
Maternal APN	0.027	0.090	-0.371	-0.271	**0.392**(*p* = 0.008)	0.027	**-0.365**(*p* = 0.015)	-0.207	**0.505** (*p* < 0.0001)	-0.137	**-0.286**(*p* = 0.047)	0.128
Maternal Leptin	**0.476** (*p* = 0.012)	0.358	0.217	-0.001	**0.361**(*p* = 0.016)	**0.416**(*p* = 0.005)	0.080	0.048	0.170	**0.586**(*p* < 0.0001)	-0.148	-0.274
Maternal Blood Glucose	0.246	0.368	0.328	-0.027	0.226	0.089	0.025	0.136	0.024	0.222	-0.033	-0.160
Maternal HbA1c	-0.180	-0.106	0.247	0.155	-0.202	0.030	-0.046	**0.404** (*p* = 0.010)	0.129	**0.332**(*p* = 0.018)	**0.386**(*p* = 0.008)	**0.444**(*p* = 0.004)

Cord blood and maternal leptin showed a strong positive correlation in T2D pregnancies (*r* = 586, *p* < 0.0001), and a moderate correlation in GDM pregnancies (*r* = 416, *p* = 0.005) but no significant correlation in control pregnancies (*p* = 0.07) (Table 2). Maternal leptin was positively correlated with cord blood adiponectin in control (*p* = 0.012) and GDM (*p* = 0.016), but not T2D pregnancies (Table 2). Maternal HbA1C only showed a positive correlation with cord blood leptin (*p* = 0.018) in T2D pregnancies (Table 2).

### Associations between birthweight and cord blood adipokines, insulin and birthweight

Cord blood leptin levels showed a weak positive correlation with birthweight z-score in GDM pregnancies (*r* = 0.348, *p* = 0.031) (Table 2). In addition, cord blood leptin was associated with increased ponderal index only in GDM pregnancies (*p* = 0.02) (Supplemental Table 4), but not control and T2D pregnancies. Cord blood adiponectin was not associated with birthweight z-score in any groups of pregnancies (Table 2). However, cord blood insulin levels were positively correlated with birthweight z-score in all groups of pregnancies (Table 2).

Cord blood leptin (*p* = 0.044), but not cord blood adiponectin, showed a positive correlation with cord blood insulin in control pregnancies (Table 2). Correlations between cord blood insulin and leptin were not observed in GDM and T2D pregnancies (Table 2). Cord blood insulin also showed a moderate positive correlation with maternal age at delivery (*p* = 0.041), parity (*p* = 0.016) and birthweight z-scores (*p* < 0.001) in control pregnancies (Table 2, Supplemental Table 4). In GDM pregnancies, cord blood insulin did not correlate with maternal age at delivery, parity or birthweight z-score (Table 2, Supplemental Table 4). In T2D pregnancies, cord blood insulin was positively correlated with gestational weight gain (*p* = 0.039) and strongly correlated with birthweight z-score (*p* < 0.0001) (Table 2, Supplemental Table 4). Cord blood insulin showed a positive correlation with maternal HbA1C in GDM (*r* = 0.404, *p* = 0.010) and T2D pregnancies (*r* = 0.444, *p* = 0.004) (Table 2).

## Discussion

The objective of this study was to determine whether there are differential impacts on maternal serum and cord blood levels of adiponectin, leptin and insulin at delivery based upon the duration of diabetes during pregnancy (GDM versus pre-existing T2D) and whether these levels are associated with fetal growth and birthweight. This is the first and largest study to report the association between infant weight and the maternal/cord blood adiponectin, leptin and insulin levels in pregnancies affected by GDM or T2D in a First Nations population. We showed that GDM and T2D had distinct maternal serum and cord blood serum adiponectin and leptin profiles.

Consistent with what is known about adipokines in pregnancy, we found that maternal adiponectin levels were lower in the GDM group. Interestingly, maternal T2D had no effect on serum adiponectin levels and were in fact significantly higher than in GDM women. Since BMI impacts adiponectin levels, we sub-grouped the data into obese and non-obese groups, even though the non-obese groups were underpowered (*N* = 5–8 per group), and there were insufficient participants with normal BMI (< 25 kg/m^2^) to perform any analysis on mothers with exclusively healthy BMI. The non-obese T2D group had significantly lower serum adiponectin levels, suggesting possible dysregulation of adiponectin secretion during pregnancy in T2D women, though this merits further study. Obesity, in the absence of diabetes, also appears to lead to decreased maternal adiponectin, which is expected given the reduced adiponectin secretion that is observed in obesity [16]. There were no differences in serum adiponectin between GDM and T2D mothers, regardless of BMI, however as mothers with T2D have experienced hyperglycemia and insulin resistance for a longer period, treatment could have impacted adiponectin levels. Individuals with T2D were diagnosed prior to enrollment and received standard nutritional and physical activity advice. Some of these individuals may have also received insulin and/or oral medication to manage their diabetes when necessary. Both in vivo and in vitro results using adipose tissue explants showed that metformin is capable of increasing adiponectin gene expression [21], and in male subjects with T2D insulin therapy significantly increased circulating adiponectin [22]. In this study, the majority of T2D mothers were treated with insulin and a smaller proportion were treated with metformin. Thus, insulin and metformin therapy may have impacted maternal serum adiponectin in this study.

As pregnancy progresses through the first and second trimester, leptin levels increase and are maintained throughout the third trimester and then decrease postpartum [17]. Obese women and women with GDM have higher serum leptin compared to women with a healthy BMI in pregnancy [13, 14]. In this study, maternal leptin was positively correlated with maternal BMI at delivery in all groups, but this was only significant in mothers with GDM. Subgroup analysis of non-obese and obese groups revealed that maternal leptin tended to be about 3-4-fold higher in obese groups, compared to non-obese groups.

Levels of adipokines in the maternal and fetal systems are implicated in fetal growth and metabolism [23]. Maternal adiponectin shows an inverse relationship with birthweight [12]. Maternal adiponectin does not cross the placenta and exert its effects on offspring by signalling through placental adiponectin receptors [17]. In contrast, there is a positive association between fetal growth and adiposity with fetal adiponectin and an inverse relationship to maternal glycemia [16]. Fetal adiponectin promotes fetal growth and potentiates fetal insulin resistance [24]. The project VIVA study found an association between increased cord blood adiponectin and increased childhood adiposity at 3 years of age [25]. We observed no correlations between birthweight and fetal adiponectin in this study, though it is possible that maternal obesity masked associations in this cohort. Similarly, Luo et al. did not report any association between maternal diabetes or BMI and cord blood adiponectin levels [24]. There are currently few studies reporting the effect of GDM and T2D on cord-blood adiponectin, however our results agree with those reported by the ACHOIS study and by Cortelazzi et al., wherein infants born to GDM mothers with obesity had lower cord-blood adiponectin [26, 27].

One study reported that neonatal leptin is higher than maternal leptin [28], however others [29] observed that cord blood leptin corresponded to maternal leptin levels, similar to our findings. Studies have shown that fetal leptin increases throughout gestation [17]. Cord blood leptin is positively correlated with birthweight, length and adiposity [17]. In our study, cord blood leptin was positively correlated with birthweight-z scores in all groups, but this was significant only in GDM pregnancies. Cord blood insulin, however, was positively correlated with birthweight z-scores across all groups. It has been postulated that leptin in pregnancy may signal changing nutrient availability in the mother, since cord blood leptin appears to be influenced by maternal diabetes and may impact the effect of insulin on fetal growth [30]. Moreover, maternal leptin positively correlated with BMI at delivery in the GDM pregnancies, suggesting that the interplay between maternal obesity and leptin influences the infant cord blood leptin and birthweight.

The matched mother-infant pairs from a First Nations population facilitates the novel comparison of maternal and cord blood adipokines in GDM and T2D pregnancies on birthweight. However, our study has several limitations. Some maternal characteristics were self-reported including medical history and smoking status. It is possible that this data was subject to recall bias (family and individual medical history) and that smoking status may have been underreported due to the stigma associated with smoking during pregnancy. However, we did have a very high proportion of mothers who reported smoking within the first trimester and throughout pregnancy which suggests that underreporting may not have been that significant of an issue. In addition, we were unable to confirm from self-report whether mothers in this study had a previous GDM diagnosis. Compared to newly diagnosed GDM, a previous GDM pregnancy is associated with a higher pre-pregnancy BMI and increased fasting blood glucose [31]. In addition, most mothers in our cohort entered pregnancy overweight or obese (Supplemental Table 1). Women who enter pregnancy at a higher BMI exhibit reduced gestational weight gain [32]. Gestational weight gain affects maternal and cord blood adipokines [33] and neonatal outcomes [32]. Therefore, obesity and altered patterns of gestational weight gain in this cohort may mask some associations. Additionally, we collected serum for measurement of maternal adipokines at term and consequently the results do not provide a picture of pregestational adipokine levels and the dynamic changes throughout pregnancy. It is important to note that mothers with T2D in our cohort were prescribed insulin or metformin to achieve glycemic control and these interventions may have affected circulating adiponectin levels and birthweight.

## Conclusions

These results show differential maternal and cord blood adipokine profiles in pregnancies with normoglycemia, GDM and pregestational T2D. Maternal adiponectin correlated with birthweight in control pregnancies, whereas maternal leptin correlated with birthweight only in T2D pregnancies. Offspring with the highest birthweight were born to mothers with T2D and birthweight was associated with higher gestational weight gain, cord blood leptin, insulin and glucose. Cord blood adipokines were impacted by maternal diabetes status and reflected other risk factors such as gestational age at delivery and birth weight. The increased risks associated with exposure to hyperglycemia in pregnancy necessitate better understanding of mechanisms underlying the distinct pathophysiology and consequences of GDM and pre-pregnancy T2D.

## Electronic supplementary material

Below is the link to the electronic supplementary material.

Supplementary Material 1

## Data Availability

Data is provided within the manuscipt and supplementary information.

## Abbreviations

BMI: Body Mass Index
CRP: C-reactive protein
GDM: Gestational Diabetes Mellitus
HbA1c: Hemoglobin A1c
SEM: Standard Error of the Mean
T2D: Type 2 Diabetes
