# Supplementary material for: Differential effects of type 2 diabetes and gestational diabetes on maternal and cord blood adipokines and newborn weight
Source: BMC Pregnancy Childbirth. 2025 Mar 5;25:238. doi: 10.1186/s12884-025-07169-z (PMC11881472; doi:10.1186/s12884-025-07169-z)
Supplement: Supplementary file 1 — Supplementary Material 1 [file 12884_2025_7169_MOESM1_ESM.docx]

**Supplemental Material**

**Differential Effects of Type 2 Diabetes and Gestational Diabetes on Maternal and Cord blood Adipokines and Newborn Weight**

Running title: Diabetes in pregnancy, adipokines and infant weight

Brittany L. Gruber^1,4^, Yash Rawal^2,4^, Priscilla Irabor^2,4^, Elizabeth A. C. Sellers^2,4^,

Christy Pylypuk^3^, Vernon W. Dolinsky^1,4,5^ & Brandy A. Wicklow^2,4,5^

1. Departments of Pharmacology & Therapeutics, 2. Pediatrics & Child Health, 3. Obstetrics & Gynecology, University of Manitoba and 4. Diabetes Research Envisioned and Accomplished in Manitoba (DREAM) Research Theme of the Children’s Hospital Research Institute of Manitoba

Authors for Co-correspondence:

^5^Dr. Brandy A. Wicklow e-mail: bwicklow@exchange.hsc.mb.ca

Associate Professor of Pediatrics & Child Health

University of Manitoba,

Research Scientist of the Diabetes Research Envisioned and Accomplished in Manitoba (DREAM) Research Theme of the Children’s Hospital Research Institute of Manitoba.

FE307-685 William Avenue

Winnipeg, MB, Canada R3E 0Z2

Telephone number: 204-789-3559

Fax Number: 204-789-3915

^5^Dr. Vernon W. Dolinsky e-mail: vdolinsky@chrim.ca

Professor of Pharmacology & Therapeutics

University of Manitoba,

Research Scientist of the Diabetes Research Envisioned and Accomplished in Manitoba (DREAM) Research Theme of the Children’s Hospital Research Institute of Manitoba.

601 John Buhler Research Centre,

715 McDermot Avenue

Winnipeg, MB, Canada R3E 3P4

Telephone number: 204-789-3559

Fax Number: 204-789-3915

Number of supplemental figures: 1

Number of Supplemental Tables: 6

| Maternal | Control | | |  | GDM | | |  | T2D | | | |  | |
| --- | --- | --- | --- | --- | --- | --- | --- | --- | --- | --- | --- | --- | --- | --- |
|  | **Mean** | **SD** | **N** | **p** | **Mean** | **SEM** | **N** | **p** | **Mean** | **SEM** | **N** | **p** | |  |
| Age @ Delivery (years) | 26.89 | 6.22 | 42.00 |  | 28.65 | 6.01 | 61.00 |  | 27.86 | 6.40 | 70.00 |  | |  |
| Parity | 1.40 | 1.70 | 35.00 |  | 3.02* | 2.44 | 60.00 | 0.001 | 2.43^#^ | 2.20 | 60.00 | 0.041 | |  |
| Gravidity | 2.97 | 2.16 | 34.00 |  | 4.65* | 1.92 | 59.00 | 0.005 | 4.26^#^ | 2.69 | 61.00 | 0.024 | |  |
| Maternal Blood Glucose (mmol/L) | 5.43 | 1.06 | 18.00 |  | 5.72 | 1.77 | 27.00 |  | 9.46^**#^ | 3.90 | 31.00 | p<0.0001  p<0.0001 | |  |
| BMI @ Enrollment | 30.10 | 6.53 | 29.00 |  | 33.26 | 6.07 | 33.00 |  | 32.08 | 6.61 | 36.00 |  | |  |
| BMI @ Delivery | 34.16^1^ | 6.47 | 37.00 | 0.027 | 35.72 | 6.43 | 58.00 |  | 35.27^1^ | 5.89 | 64.00 | 0.038 | |  |
| Smoking % | 46.34% | 50.49% | 41.00 |  | 50.82% | 50.41% | 61.00 |  | 47.83% | 50.32% | 69.00 |  | |  |
| GWG (Kg) | 12.23 | 6.69 | 29.00 |  | 8.01* | 6.04 | 30.00 | 0.024 | 12.36** | 5.52 | 34.00 | 0.014 | |  |
| CRP | 8.20 | 5.33 | 40.00 |  | 11.38 | 15.46 | 60.00 |  | 7.49 | 5.95 | 61.00 |  | |  |
| HbA1c (%) | 5.43 | 0.48 | 40.00 |  | 5.99* | 1.05 | 61.00 | 0.003 | 7.54**^#^ | 1.55 | 64.00 | <0.0001, <0.0001 | |  |
|  |  |  |  |  |  |  |  |  |  |  |  |  | |  |

**Supplemental Table 1:** Supplemental Table 1: Maternal characteristics in the Next Generation Cohort used for adiponectin and leptin analysis. Data represents mean, ± Standard deviation. Statistical significance established by p<0.05, p-values reported for comparisons as follows: * control vs GDM, **GDM vs T2D, # control vs T2D, ^1^enrollment vs delivery. P-values determined by One-way ANOVA or Kruskal-Wallis test where appropriate. N represents individual mothers (unpaired). CRP: C-reactive protein, GWG: Gestational weight gain, HbA1c: Hemoglobin A1c (%).

| Neonatal | Control | | |  | GDM | | |  | T2D | | |  |
| --- | --- | --- | --- | --- | --- | --- | --- | --- | --- | --- | --- | --- |
|  | **Mean** | **SD** | **N** | **P** | **Mean** | **SD** | **N** | **P** | **Mean** | **SD** | **N** | **P** |
| Male Length (cm) | 53.39 | 2.99 | 12 |  | 50.49* | 1.99 | 35 | 0.024 | 50.24^#^ | 3.75 | 24 | 0.019 |
| Female Length (cm) | 50.81 | 2.95 | 18 |  | 49.76 | 2.46 | 22 |  | 49.75 | 2.60 | 45 |  |
| Male Length z-score | 1.75 | 1.41 | 13 |  | 1.252 | 0.99 | 34 |  | 0.89 | 1.68 | 26 |  |
| Female Length z-score | 1.17 | 1.43 | 19 |  | 1.212 | 1.42 | 22 |  | 1.49 | 1.40 | 45 |  |
| Proportion Male | 58.1% |  | 18 |  | 39.0% |  | 37 |  | 41.8% |  | 33 |  |
| Male Birthweight (g) | 3617 | 735.6 | 15 |  | 3420 | 488.6 | 37 |  | 3328 | 814.5 | 31 |  |
| Female Birthweight g | 3377 | 450.4 | 17 |  | 3331 | 528.1 | 24 |  | 3522 | 491.1 | 49 |  |
| Male Birthweight z-score | 0.820 | 1.11 | 15 |  | 0.871 | 1.05 | 37 |  | 0.817 | 1.39 | 31 |  |
| Female Birthweight z-score | 0.424 | 0.94 | 19 |  | 0.993** | 1.03 | 24 | 0.031 | 1.71^#$^ | 1.11 | 49 | 0.0001,  0.002 |
| Male birthweight percentile | 70.13 | 26.9 | 15 |  | 76.22 | 22.9 | 36 |  | 67.43^$^ | 34.1 | 31 | 0.005 |
| Female Birthweight percentile | 61.19 | 25.6 | 19 |  | 75.23 | 24.4 | 24 |  | 83.18^#^ | 21.0 | 51 | 0.003 |
| Male Gestational age (weeks) | 39.10 | 2.02 | 15 |  | 37.68* | 1.19 | 37 | 0.004 | 37.25^#^ | 1.98 | 32 | 0.0002 |
| Female Gestational age (weeks) | 38.96 | 0.98 | 18 |  | 37.50* | 1.05 | 24 | 0.004 | 36.85^#^ | 1.27 | 51 | <0.0001 |
| Male Ponderal Index (g/cm^3^) | 2.55 | 0.32 | 14 |  | 2.65 | 0.32 | 35 |  | 2.59 | 0.32 | 25 |  |
| Female Ponderal Index (g/cm^3^) | 2.53 | 0.32 | 19 |  | 2.76 | 0.38 | 21 |  | 2.89^$#^ | 0.42 | 45 | 0.002,  0.003 |

**Supplemental Table 2:** Neonatal characteristics of infants in the Next Generation Cohort used for adiponectin and leptin analysis. Data represents mean, ± Standard deviation. Statistical significance established where p<0.05 as determined by One-way ANOVA or Kruskal-Wallis test where appropriate. P-values reported for comparisons described, as follows: *control vs GDM, **GDM vs T2D, # control vs T2D, $ male vs female. N represents neonates only, independent of maternal data (unpaired). GDM, Gestational diabetes mellitus; T2D, Type-2 diabetes; BW, birthweight, GA, Gestational age.

|  | Control | | GDM | | T2D | |
| --- | --- | --- | --- | --- | --- | --- |
|  | Maternal APN | Maternal LEP | Maternal APN | Maternal LEP | Maternal APN | Maternal LEP |
| Parity | -0.158 | **-0.477 *(p=0.021)*** | **0.318 *(p=0.033)*** | -0.099 | -0.214 | 0.118 |
| Ponderal Index | 0.050 | -0.247 | 0.047 | 0.106 | -0.147 | -0.113 |
| Birthweight z-score | -0.371 | 0.217 | **-0.365 *(p=0.015)*** | 0.080 | **-0.286 *(p=0.047)*** | -0.148 |
| Length z-score | -0.390 | 0.217 | **-0.514 *(p<0.0001*)** | 0.056 | -0.041 | **-0.375 (p=0.012)** |
| Maternal CRP | -0.378 | **0.517 *(p=0.006)*** | -0.196 | 0.046 | **-0.309 *(p=0.032)*** | 0.234 |
| GWG | 0.120 | -0.343 | 0.018 | 0.007 | 0.320 | -0.144 |
| BMI @ Enrollment | **-0.544 *(p=0.011)*** | 0.304 | -0.018 | 0.246 | -0.116 | 0.006 |

**Supplemental Table 3** Correlations between maternal adipokines and neonatal outcomes in paired mother-infant dyads from control, GDM and T2D pregnancies. Values represent Spearman’s correlation coefficient (r value) from regressions performed on maternal and cord blood adipokine and serum measures against maternal and fetal anthropometric values. Bold font indicates statistical significance as determined by p<0.05 from Spearman correlation analysis. Italics indicates approximate p-value, computed for non-parametric regression with >18 pairs. GDM, Gestational diabetes mellitus; T2D, Type 2 diabetes mellitus; APN, adiponectin; LEP, leptin; GWG, gestational weight gain; BMI, body mass index; CRP, C-reactive protein.

|  | Control | | | | GDM | | | | T2D | | | |
| --- | --- | --- | --- | --- | --- | --- | --- | --- | --- | --- | --- | --- |
|  | Cord-blood APN | Cord-blood Leptin | Cord-blood  Insulin | Birthweight z-score | Cord-blood APN | Cord-blood Leptin | Cord-blood  Insulin | Birthweight z-score | Cord-blood APN | Cord-blood Leptin | Cord-blood  Insulin | Birthweight z-score |
| Parity | -0.14 | -0.02 | **0.53 *(p=0.016)*** | 0.23 | -0.06 | -0.06 | 0.26 | -0.24 | 0.10 | 0.14 | -0.19 | -0.12 |
| Ponderal Index | 0.10 | 0.23 | 0.12 | 0.30 | 0.10 | **0.34 *(p=0.020)*** | 0.11 | **0.63 *(p<0.0001)*** | 0.03 | 0.19 | 0.21 | **0.36 *(p=0.015)*** |
| Maternal CRP | -0.03 | 0.14 | -0.13 | 0.30 | -0.08 | 0.17 | -0.03 | 0.16 | -0.003 | 0.03 | -0.26 | -0.07 |
| GWG | 0.19 | 0.10 | -0.04 | 0.08 | 0.11 | -0.03 | -0.21 | 0.22 | 0.08 | 0.22 | **0.46 *(p=0.028)*** | 0.24 |
| BMI @ Enrollment | -0.14 | -0.003 | 0.18 | **0.44 *(p=0.045)*** | 0.18 | -0.04 | **-0.48 *(p=0.025)*** | 0.10 | 0.02 | -0.18 | -0.05 | 0.15 |
| Age @ Delivery | -0.18 | -0.14 | **0.44 *(0.041)*** | 0.12 | -0.11 | -0.001 | 0.05 | -0.13 | -0.08 | 0.10 | -0.27 | 0.002 |

**Supplemental Table 4:** Correlations between cord blood adipokines and insulin and neonatal outcomes from paired mother-infant dyads from control, GDM and T2D pregnancies. Values represent Spearman’s correlation coefficient (r value) from regressions performed on maternal and cord blood adipokine and serum measures against maternal and fetal anthropometric values. Bold font indicates statistical significance as determined by p<0.05 from Spearman correlation analysis. Italics indicates approximate p-value, computed for non-parametric regression with >18 pairs. GDM, Gestational diabetes mellitus; T2D, Type 2 diabetes mellitus; APN, adiponectin; LEP, leptin; GWG, gestational weight gain; BMI, body mass index; CRP, C-reactive protein.

|  | Control | | GDM | | T2D | |
| --- | --- | --- | --- | --- | --- | --- |
|  | Maternal APN | Maternal LEP | Maternal APN | Maternal LEP | Maternal APN | Maternal LEP |
| Parity | -0.158 (-0.54-0.28) | **-0.477 (-0.75 –**  **-0.07)** | **0.318 (0.02-0.57)** | -0.100 (-0.39-0.21) | -0.214 (-0.48-0.08) | 0.120 (-0.18-0.40) |
| Birthweight z-score | -0.371 (-0.68-0.05) | 0.217 (-0.22-0.58) | **-0.365 (-0.60-**  **-0.07)** | 0.081 (-0.23-0.38) | **-0.286 (-0.53-**  **-0.003)** | -0.148 (-0.42-0.15) |
| Length z-score | -0.390 (-0.70-0.04) | 0.217 (-0.23-0.59) | **-0.514 (-0.71-**  **-0.25)** | 0.056 (-0.25-0.35) | -0.041 (-0.34-0.26) | -0.375 (-0.61-0.08) |
| Maternal CRP | -0.378 (-0.67-0.01) | **0.517 (0.16-0.75)** | -0.196 (-0.47-0.11) | 0.046 (-0.26-0.36) | **-0.309 (-0.55 –**  **-0.02)** | 0.234 (-0.06-0.49) |
| GWG | 0.120 (-0.35-0.54) | -0.343 (-0.70-0.13) | 0.018 (-0.40-0.41) | 0.007 (-0.40-0.41) | 0.320 (-0.07-0.63) | -0.144 (-0.51-0.27) |
| BMI @ Enrollment | **-0.544 (-0.80 -**  **-0.13)** | 0.304 (-0.16-0.66) | -0.018 (-0.40-0.37) | 0.246 (-0.17-0.59) | -0.116 (-0.47-0.27) | 0.006 (-0.38-0.39) |
| BMI @ Delivery | **-0.545 (-0.79 –**  **-0.16)** | 0.409 (-0.02-0.71) | -0.253 (-0.52-0.05) | **-0.371 (0.07-0.61)** | -0.037 (-0.31-0.25) | 0.207 (-0.08-0.46) |
| Maternal Age | -0.131 (-0.49- 0.27) | -0.208 (-0.60-0.12) | 0.110 (-0.19-0.39) | 0.006 (-0.30-0.31) | **-0.388 (-0.60 –**  **-0.13)** | 0.178 (-0.10-0.43) |
| HbA1c | -0.229 (-0.58-0.20) | -0.021 (-0.42-0.39) | 0.070 (-0.23-0.36) | 0.222 (-0.09-0.49) | 0.058 (-0.24-0.34) | 0.147 (-0.15-0.42) |

**Supplemental Table 5**: Correlations between maternal serum adipokines, and neonatal and maternal characteristics in paired mother-infant dyads from control, GDM and T2D pregnancies. Values represent Spearman’s correlation coefficient (r value) from regressions performed on maternal and cord blood adipokine and serum measures against maternal and fetal anthropometric values. Bold font indicates statistical significance as determined by p<0.05 from Spearman correlation analysis, with 95% confidence intervals of r (in brackets). GDM, Gestational diabetes mellitus; T2D, Type 2 diabetes mellitus; APN, adiponectin; LEP, leptin; GWG, gestational weight gain; BMI, body mass index; CRP, C-reactive protein.

|  | Control | | | GDM | | | T2D | | |
| --- | --- | --- | --- | --- | --- | --- | --- | --- | --- |
|  | Cord-blood APN | Cord-blood Leptin | Cord-blood  Insulin | Cord-blood APN | Cord-blood Leptin | Cord-blood  Insulin | Cord-blood APN | Cord-blood Leptin | Cord-blood  Insulin |
| Birthweight z-score | 0.22 (-0.22-0.59) | 0.35 (-0.10-0.68) | **0.70 (0.37-0.87)** | -0.01 (-0.30-0.29) | **0.32 0.02-0.56)** | **0.33 (0.01-0.60** | -0.14 (-0.41-0.16) | 0.18 (-0.12-0.44) | **0.74 (0.56-0.86)** |
| Length z-score | -0.02 (-0.44-0.42) | 0.24 (0.23-0.62) | **0.70 (0.36-0.88)** | -0.14 (-0.41-0.18) | 0.10 (-0.21-0.38) | **0.30 (-0.03-0.56)** | -0.11 (-0.40-0.19) | 0.02 (-0.32-0.29) | **0.64 (0.40-0.80)** |
| BMI @ Delivery | -0.03 (-0.45-0.39) | 0.12 (-0.33-0.52) | 0.14 (-0.34-0.56) | -0.07 (-0.36-0.24) | 0.10 (-0.22-0.38) | 0.09 (-0.24-0.40) | 0.20 (-0.09-0.45) | 0.11 (-0.17-0.38) | 0.18 (-0.13-0.43) |
| BMI @ Enrollment | -0.14 (-0.56-0.34) | -0.003 (-0.46-0.45) | 0.18 (-0.32-0.59) | 0.18 (-0.22-0.52) | -0.04 (-0.41-0.35) | **-0.48 (-0.75- -0.05)** | 0.02 (-0.36-0.40) | -0.18 (-0.53-0.22) | 0.01 (-0.42-0.44) |
| Maternal HbA1c | -0.18  (-0.55-0.24) | -0.11 (-0.49-0.32) | 0.16 (-0.31-0.56) | -0.20 (-0.47-0.10) | 0.03 (-0.27-0.33) | **0.40 (0.10-0.64)** | 0.13 (-0.17-0.40) | **0.33 (0.05-0.56)** | **0.44 (0.14-0.67)** |
| Maternal APN | 0.03 (-0.37-0.41) | 0.10 (-0.32-0.47) | -0.29 (-0.63-0.16) | **0.32 (0.10-0.62)** | 0.03 (-0.28-0.33) | -0.21 (0.50-0.13) | **0.51 (0.26-0.70)** | -0.13 (-0.40-0.15) | 0.12 (-0.20-0.40) |
| Maternal Leptin | **0.48 (0.10-0.73)** | 0.36 (-0.05-0.66) | -0.001 (-0.43-0.43) | **0.36 (0.06-0.60)** | **0.42 (0.13-0.64)** | 0.05 (-0.23-0.42) | 0.17 (-0.11-0.43) | **0.59 (0.37-0.74)** | -0.27 (-0.54-0.04) |
| Cord-blood APN |  |  |  |  |  |  |  |  |  |
| Cord-blood Leptin | **0.60 (0.27-0.81)** |  |  | 0.27 (-0.03-0.52) |  |  | **0.34 (0.07-0.57)** |  |  |
| Cord-blood insulin | 0.33 (-0.13-0.67) | **0.44 (0.001-0.74)** |  | -0.27 (-0.54-0.06) | 0.17 (-0.15-0.46) |  | 0.10 (-0.22-0.39) | 0.25 (-0.06-0.51) |  |

**Supplemental Table 6**: Correlations between cord blood adipokines and insulin and neonatal outcomes in paired mother-infant dyads from control, GDM and T2D pregnancies. Values represent Spearman’s correlation coefficient (r value) from regressions performed on maternal and cord blood adipokine and serum measures against maternal and fetal anthropometric values. Bold font indicates statistical significance as determined by p<0.05 from Spearman correlation analysis, with 95% confidence intervals of r (in brackets). GDM, Gestational diabetes mellitus; T2D, Type 2 diabetes mellitus; APN, adiponectin; LEP, leptin; GWG, gestational weight gain; BMI, body mass index; CRP, C-reactive protein.

**
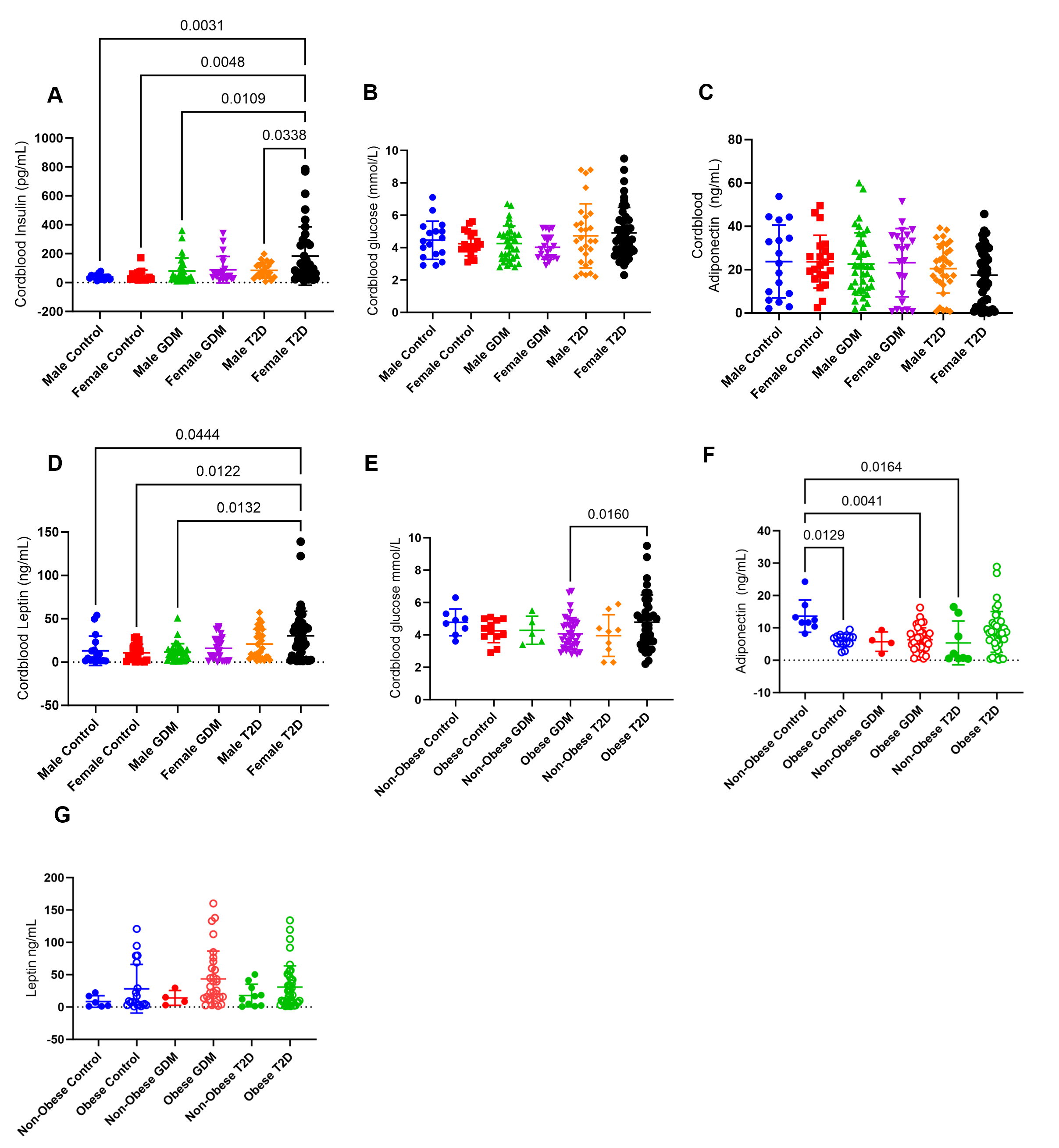
**

**Supplemental Figure 1:** Supplemental Figure 1: (A) Cord blood insulin from all neonates (Male control n=14, female control n=15, male GDM n=29, female GDM n=21, male T2D n=22, female T2D n=40), (B) cord blood glucose from all neonates (Male control n=17, female control n=18, male GDM n=34, female GDM n=22, male T2D n=28, female T2D n=47), (C) cord blood adiponectin from all neonates (Male control n=17, female control n=21, male GDM n=37, female GDM n=23, male T2D n=32, female T2D n=52), (D) and cord blood leptin from all neonates (Male control n=15, female control n=20, male GDM n=35, female GDM n=23, male T2D n=31, female T2D n=50), separated by neonatal sex. E) Cord blood glucose from paired maternal-infant dyads, stratified by maternal obesity status (Non-obese control n=8, obese control n=12, non-obese GDM n=5, obese GDM n=38, non-obese T2D n=9, obese T2D n=38). F) Maternal adiponectin from paired maternal-infant dyads, striated by maternal obesity (Non-obese control n=8, obese control n=14, non-obese GDM n=4, obese GDM n=38, non-obese T2D n=8, obese T2D n=43). G) Maternal leptin from paired maternal-infant dyads, striated by maternal obesity (Non-obese control n=6, obese control n=19, non-obese GDM n=4, obese GDM n=30, non-obese T2D n=9, obese T2D n=41).
